# On the (In)Equality of Droplet Rebound Dynamics at Fixed Weber Number

**DOI:** 10.3390/biomimetics11090640

**Published:** 2026-09-06

**Authors:** Jure Berce, Iztok Golobič

**Affiliations:** Faculty of Mechanical Engineering, University of Ljubljana, Aškerčeva 6, SI-1000 Ljubljana, Slovenia; jure.berce@fs.uni-lj.si

**Keywords:** droplet rebound, superhydrophobic surface, Weber number, rebound dynamics, maximum spreading coefficient, contact time

## Abstract

The similarity of droplet impacts on nature-mimicking superhydrophobic surfaces is traditionally compared using the dimensionless Weber number. Yet, maintaining a constant *We* by decoupling droplet diameter and impact velocity influences secondary forces, challenging this assumption of similarity. In this work, we investigate water droplet impacts on a lotus-leaf-mimicking laser-textured superhydrophobic aluminum surface at two constant Weber number levels (25 and 50), varying droplet diameter from 2.1 to 4.15 mm. Our results confirm that maximum spreading depends on the Reynolds number at a fixed *We*, as smaller, faster droplets spread less due to increased relative viscous dissipation. We propose a modified empirical scaling model that describes our data with high accuracy and generalizes successfully to external datasets. Crucially, we demonstrate that the contact time of a droplet of a given size is not strictly velocity-independent, unveiling a Weber number-dependent inertia-capillary scaling. We show that this is driven by a shift in rebound dynamics, where the relative timescale of spreading increases over retraction for larger droplets. These findings demonstrate that *We* is insufficient to characterize droplet rebound across varying scales and that accounting for size-dependent deviations is critical for the precise design of technologies that leverage droplet-surface interactions.

## 1. Introduction

The phenomenon of liquid droplets impacting solid surfaces governs the efficiency of many natural and industrial processes, from aerodynamic accumulation of ice on aircraft wings to precision of inkjet printing and spray cooling, as well as targeted delivery of agricultural pesticides. In nature, biological surfaces have evolved intricate multi-scale structures to maintain water repellency, self-cleaning, and structural durability under droplet impacts ranging from fine fog droplets to heavy rainfall. Deciphering how these interfaces dissipate and transform energy during liquid–solid contact provides the fundamental framework for developing artificial biomimetic surfaces. When a droplet impacts a superhydrophobic surface (an interface with low surface energy imparted on micro- or nanoscale roughness [1] to achieve a contact angle over 150° with a hysteresis below 10° [2]), it typically undergoes a complex shape evolution consisting of spreading, retraction, and rebound. Depending on the initial energy and its transformation, the collision may result in adhesion, complete rebound, partial rebound, or fragmentation and splashing.

In classic interfacial fluid dynamics, the outcomes of a droplet impact are predominantly categorized and predicted using dimensionless numbers that express the ratios of competing physical forces. The most critical of these are the Weber and Reynolds numbers, defined as $We=\rho {v_{0}}^{2}D_{0}/\sigma$ and $Re=\rho v_{0}D_{0}/\mu$ where *ρ* is the liquid density, *v*_0_ the impact velocity, *D*_0_ the droplet diameter, *σ* the surface tension of the liquid, and *μ* its dynamic viscosity. The parameters quantify the relative importance of the droplet’s inertia compared to capillarity and viscosity, respectively, while the impact regimes are traditionally delineated under the assumption that impacts sharing the same dimensionless numbers will exhibit dynamically similar behaviors. The latter allows researchers to extrapolate laboratory observations to large-scale industrial applications.

At low impact velocities, where the surface energy of the droplet is larger than the kinetic energy (*We* < 1), the droplet spreads only slightly and remains relatively spherical, whereas at high impact velocities (*We* ≥ 1), the deformation of the droplet is significant [3]. In the latter case, upon impact, the droplet resembles a truncated sphere and a lamella begins to eject from the droplet, forming a thin film bounded by a rim [4]. Droplet spreading continues until a maximum is reached, which is commonly reported in the form of a maximum droplet diameter *D*_max_ or a nondimensional maximum spreading coefficient $\beta_{max}=D_{max}/D_{0}$. The droplet spreading dynamics, as well as its maximal radial extension, depend on the nature of the rebound and can fall into the inertia and viscous regime, based on the value of the impact number $P=We/{Re}^{4/5}$ [5]. In the inertial regime (*P* < 1), the maximum droplet spread mainly depends on the balance between inertia and capillarity and scales as *We*^1/4^, while in the viscous regime (*P* > 1), the spreading is limited by the effect of viscosity, and the maximum spread scales as *Re*^1/5^.

After the droplet retracts back towards the initial shape, it begins to extend vertically up to the point where it eventually breaks contact with the surface. Although the rebound consists of several complex phenomena, the whole process lasts for only a few short moments, as the contact time between a surface and a millimetric-sized water droplet is measured at approximately 10 milliseconds [6]. The time needed for an inviscid drop to rebound off a surface scales with the inertia-capillary timescale $t_{ic}={\left(\rho {R_{0}}^{3}/\sigma \right)}^{1/2}$ and is independent of the impact velocity. Yet, the latter has a significant effect on the efficiency of rebound, described by the coefficient of restitution $\varepsilon =v_{reb}/v_{0}$, where *v*_reb_ is the droplet’s velocity immediately after rebound. At low Weber numbers, the coefficient of restitution is near unity, as the deformation is negligible and, with that, also the dissipation of energy [7]. As the Weber number increases, the efficiency of rebound is reduced due to increased bulk viscous dissipation, contact angle hysteresis, internal oscillations (vibrational modes), and the excess surface energy of the droplet at take-off [3].

However, describing a given liquid’s impact dynamics through the lens of Weber number represents a significant oversimplification, especially when evaluating scenarios where a constant *We* is achieved by varying the impact velocity while inversely adjusting the droplet diameter. This decoupling alters the balance of secondary forces within the fluid, including viscous dissipation, aerodynamic drag, gravitational flattening, and localized impalement pressures. Thus, small droplets are subjected to significantly higher relative viscous dissipation than large droplets exhibiting the exact same *We*. A ten times smaller droplet will also exhibit a tenfold increase in dynamic pressure $p_{d}\approx 0.5\rho {v_{0}}^{2}$, resulting from the required increase in velocity to keep *We* constant. While recent studies have recognized the complex scaling of droplet impacts by exploring effects like spherical surface curvature [8] or achieving rebound trajectory regulation with patterned wettability [9], the consequences of decoupling droplet size and velocity at a constant *We* on a uniform superhydrophobic surface remain underexplored. Specifically, the assumption that contact time remains velocity-independent requires rigorous validation under these decoupled conditions.

This study adopts a systematic experimental campaign to uncouple the parameters of water droplet size and impact velocity, revealing their distinct effects on droplet impact dynamics at two constant Weber number levels. By analyzing spreading and retraction timescales on a laser-textured surface mimicking biological topographies, we demonstrate that *We* alone is insufficient to predict the fate of an impinging droplet across varying physical scales. Furthermore, by theoretically evaluating the kinematics of both rebound phases, we show that the assumption of velocity-independent contact time breaks down, revealing a shift in the relative importance of spreading and retraction as the size of the droplet increases. These findings offer insights for translating nature’s water-shedding mechanisms into engineered biomimetic surfaces in technologies such as anti-icing coatings, self-cleaning surfaces, and precision agricultural sprays.

## 2. Materials and Methods

### 2.1. Surface Preparation

**Figure 1:**
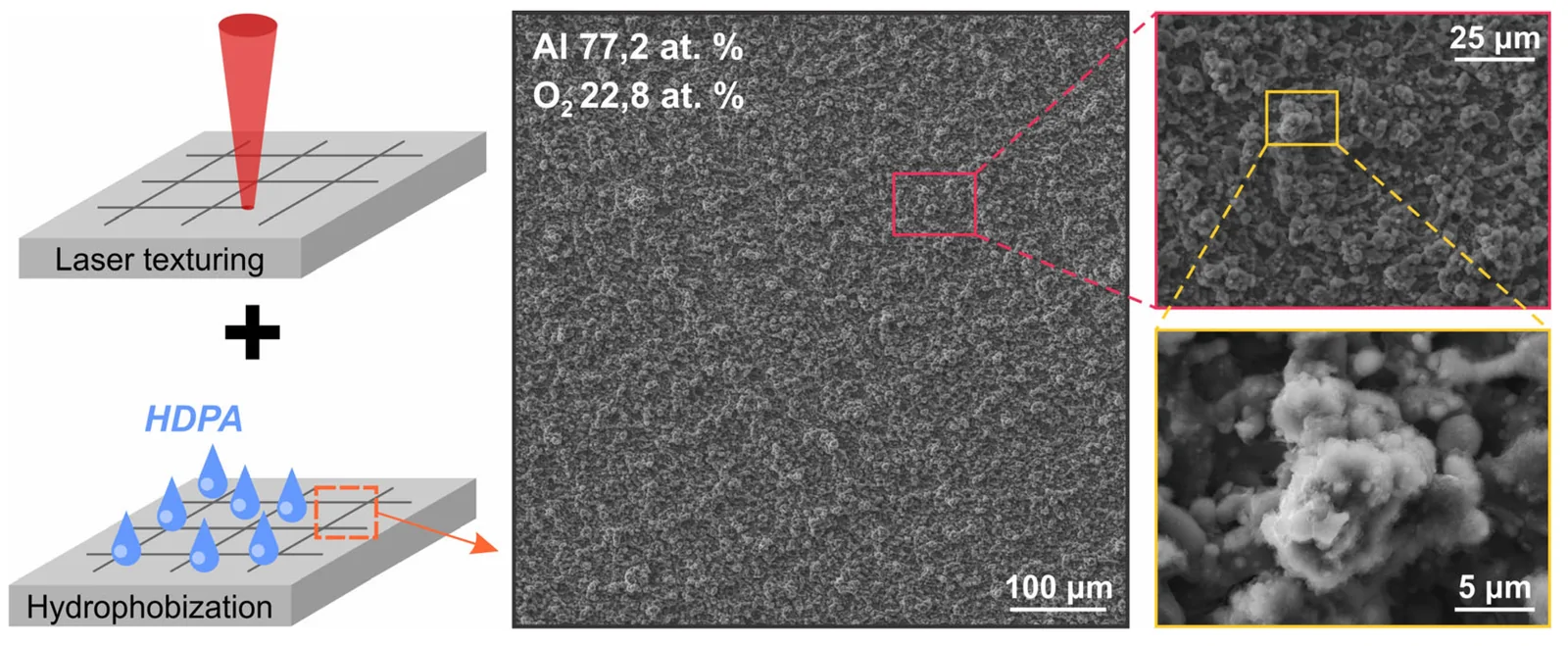
Preparation of a superhydrophobic aluminum surface with corresponding microtopography and surface chemistry.

The superhydrophobic aluminum surface was prepared using laser micromachining and an application of a hydrophobic agent (see Figure 1). An aluminum plate (1050A H24), measuring 38 × 38 mm^2^ with a thickness of 3 mm, was used as a substrate and was first ultrasonically cleaned in isopropyl alcohol (IPA) for 10 min prior to surface treatment. To impart micro- and nanoroughness, a nanosecond fiber laser was used (JPT Opto-electronics Co., Ltd., Shenzhen, China, M7 30 W MOPA; *λ* = 1064 nm; M^2^ < 1.3). An F-Theta lens with a 70 × 70 mm^2^ working area and a focal distance of 100 mm was used to guide the laser with an approximate focal beam diameter of 25 μm. Applying 60% of nominal power (*P* = 18 W), a scanning line velocity of 1650 mm s^−1^, a pulse frequency of 110 kHz and a pulse duration of 45 ns yielded an average laser pulse fluence of 33.3 J cm^−2^. With these parameters, the sample was irradiated in a 90° crosshatch pattern (scanning line separation 10 μm, inducing high overlap between neighboring passes) with a single laser beam pass to melt the upper surface layer, creating high-energy oxides and removing contaminants, resulting in a uniform morphology without any distinct surface feature orientation.

Following laser texturing, the surface was ultrasonically cleaned in IPA for 10 min in a downward-facing position to remove possible contaminants and any loosely attached re-solidified material. Afterwards, the surface was dried and cleaned using a UV/ozone cleaner (Ossila Ltd., Sheffield, UK) for 10 min, then immersed in a 3 mM solution of hexadecylphosphonic acid (HDPA; C_16_H_35_O_3_P; abcr GmbH, Karlsruhe, Germany) in ethanol at 60 °C for 1 h. Then, the sample was left to dry in ambient air and thermally annealed in a convection oven at 200 °C for 2 h to improve the durability of the formed self-assembled monolayer [10] that was imparted to the surface. The latter reduced the surface energy and ensured heterogeneous wetting with low adhesion [11], while the surface microtopography mimics the micro-papillae of the lotus leaf [12]. Such bio-inspired features promote air trapping within the cavities, enabling stable Cassie–Baxter wetting, which is important for achieving droplet rebound without premature impalement.

### 2.2. Surface Analysis

A scanning electron microscope (SEM; JEOL JCM-7000 NeoScope, Tokyo, Japan, operated at 15 kV) with energy-dispersive X-ray spectroscopy (EDS) was used to characterize the surface topography and chemistry, respectively (Figure 1). One can clearly see the stochastically distributed features in the order of <10 μm, resulting from hydrodynamic motion and ejection of melted material and its subsequent solidification. Additionally, surface wettability was evaluated at five locations using a custom-made goniometer, obtaining advancing, receding and apparent contact angles of 162.9° ± 2.9°, 156.8° ± 2.7° and 159.9° ± 1.8°, respectively, proving that the HDPA monolayer was successfully formed.

### 2.3. Experimental Procedure

**Figure 2:**
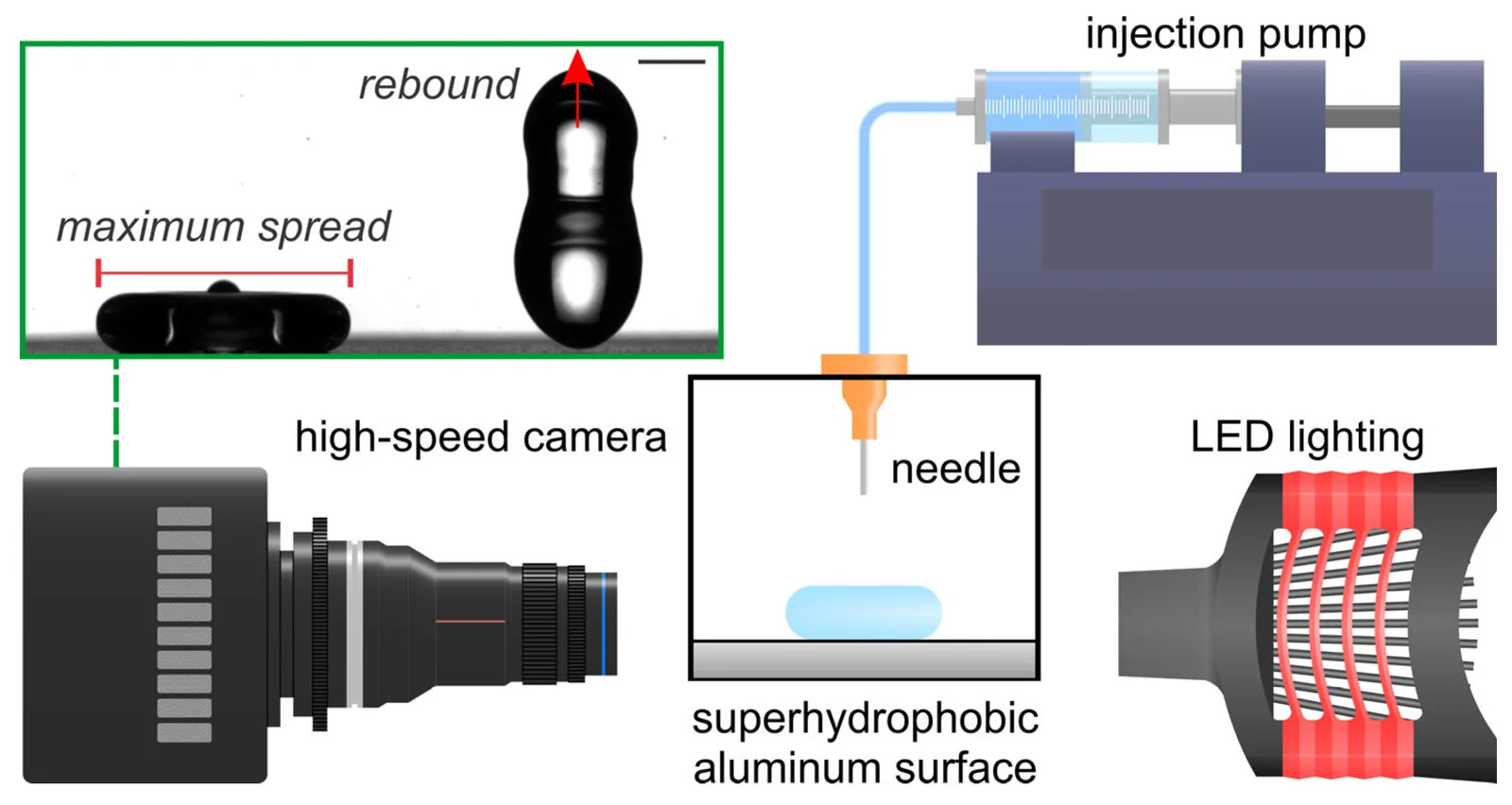
Schematic of the experimental setup used to capture droplet rebound dynamics. The figure inset shows representative snapshots at the moment of maximum spread and rebound; the scale bar corresponds to 1 mm.

De-ionized water (25 °C, *ρ* = 997.24 kg m^−3^, *μ* = 0.944 mPas, *σ* = 72.46 mN m^−1^) droplet impacts and subsequent rebounds were evaluated on a custom-designed experimental setup shown schematically in Figure 2. The setup featured a sample positioning platform and several mounts to place differently sized needles (sizes between G14 and G34) at predetermined heights above the sample to achieve the target impact velocity or droplet diameter. The impacts were recorded with a Photron Mini UX-100 (San Diego, CA, USA) high-speed camera with a macro lens (Laowa, Hefei, China, 100 mm f/2.8 2× Ultra Macro APO) at 5000 frames per second, using a strong LED backlight (Thorlabs, Newton, NJ, USA, SOLIS-2C). The droplets were created using an injection pump (Harvard Apparatus, Holliston, MA, USA, PHD 2000), and their impacts were recorded at a spatial resolution of ~19.7 μm px^−1^.

**Figure 3:**
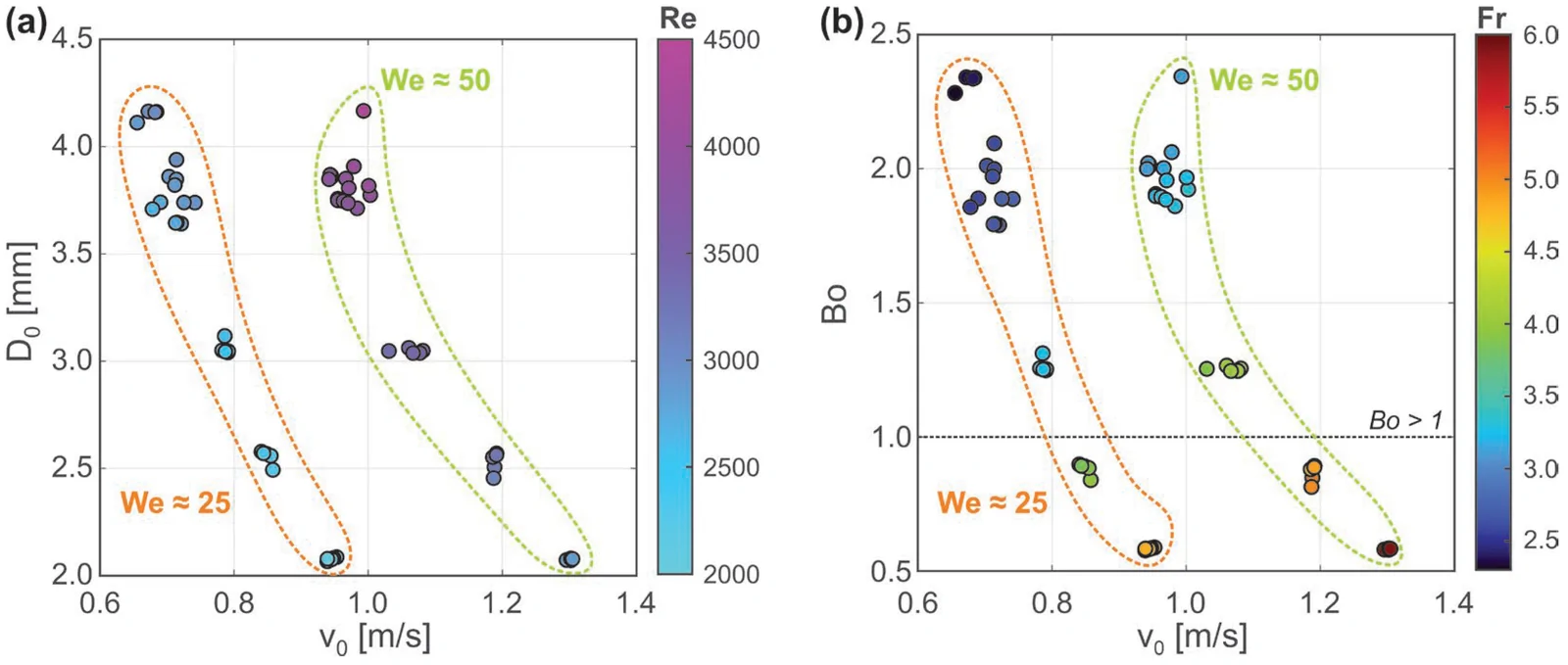
Experimental conditions: (**a**) The inverse relationship between droplet diameter and impact velocity, required to maintain two constant *We* levels, with the color scale indicating the corresponding change in *Re*. (**b**) Bond number plotted against impact velocity, showing that larger droplets cross the gravity-influenced shape threshold (*Bo* > 1), with the color scale indicating the Froude number, which remains strictly in the inertia-dominated regime (2.3 < *Fr* < 6).

To perform each measurement, the needle was positioned over the surface at the desired height, and a droplet was slowly formed by inflation at an infusion rate of 150 μL min^−1^. The droplet was left to detach naturally to ensure repeatable droplet diameter and volume. After the impact was recorded, the sample was moved in the XY direction under the syringe so that the next impact happened on a different part of the surface, avoiding potentially entrapped liquid in the microstructure from affecting the measurements. For each needle size, the measurement was repeated 5 times for each corresponding height to reach *We* number levels ~25 and ~50. The resulting matrix of experimental conditions clearly displays the required increase in velocity when *D*_0_ is reduced to maintain a constant *We* (Figure 3a). Increasing the droplet diameter above the capillary length of water (~2.7 mm) produces $Bo=\rho g{D_{0}}^{2}/\sigma$ values over 1, which indicates that the droplet shape prior to impact is influenced by gravity (Figure 3b). Yet, during rebound, the parameter describing the influence of gravity for our case is the nondimensional Froude number $Fr=v_{0}/\sqrt{gD_{0}}$. For all considered cases the *Fr* > 1 indicates that inertia plays a dominant role compared to gravity [13]. Additionally, the chosen parameter range puts all rebounds firmly in the inertia-dominated regime with values of the impact number *P* between 0.04 and 0.08, meaning that the influence of viscous dissipation in the liquid is subdominant.

### 2.4. Data Reduction and Measurement Uncertainty

Each recording of droplet impact was processed with an in-house developed algorithm in MathWorks MATLAB R2024b, explained in detail in a previous publication [14]. Briefly, the surface and droplet contours were extracted with successive steps of image subtraction, threshold-based binarization and edge detection. Immediately prior to the droplet-interface contact, the velocity *v* and diameter of the droplet *D*_0_ were recorded. The latter was computed by obtaining the droplet pixel area, revolving it around its center axis and calculating the equivalent diameter as $D_{0}≅{\left(6V/\pi \right)}^{1/3}$ to avoid the influence of gravity-induced deformation and possible oscillations of larger drops. During contact, the maximum spreading coefficient $\beta_{max}=D_{max}/D_{0}$ was computed, and once the droplet detached from the surface, marking the end of the first rebound event, the contact time *τ*_c_ was recorded. Subsequent rebound events were not recorded, while the energy efficiency (i.e., restitution coefficient) was not calculated due to differences in the number, size and velocity of satellite droplets that pinch off, making a fair comparison of energy conservation at different conditions challenging.

Standard measurement uncertainties of key parameters were evaluated by accounting for both statistical variations (Type A) and system limitations (Type B) of the setup and image processing routine. The latter included factors such as the camera frame rate, spatial discretization, and motion blur. Consequently, the maximum relative standard measurement uncertainty for the droplet diameter and impact velocity was calculated to be 0.4% and 1%, respectively. These variables, combined with minor fluid property variations caused by ambient temperature, resulted in a 3.1% relative standard measurement uncertainty for the Weber number. The propagation of uncertainty to the maximum spreading coefficient produced a relative uncertainty of 1.6%. Finally, the uncertainty of contact time, which depends on the camera’s temporal resolution to pinpoint the exact moments of contact and detachment, was evaluated at 0.08 ms.

## 3. Results and Discussion

The isotropic surface microtopography and wetting properties allowed us to avoid the influence of microstructure orientation on rebound dynamics, which would occur if larger features (e.g., microchannels) were laser-fabricated [15], which could influence the symmetry of the rebound event [16]. Additionally, due to low contact angle hysteresis (~6.1°), we can postulate that the latter’s contribution to energy loss during spreading and retraction is subdominant to other dissipation mechanisms.

### 3.1. Droplet Spreading

**Figure 4:**
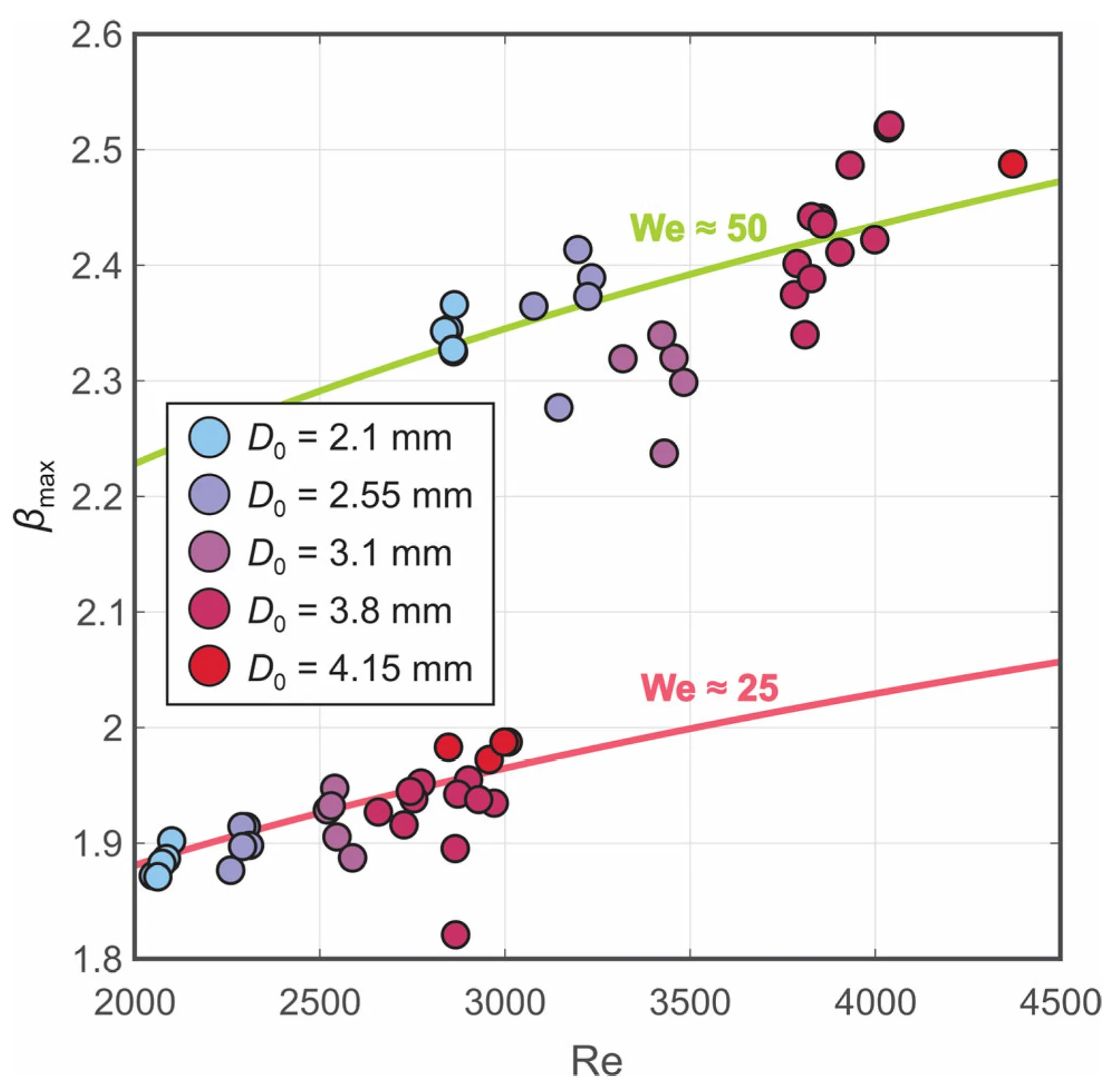
Dependence of the maximum spreading coefficient at a constant *We* number on the *Re* number (resulting from different droplet diameters). Data for each *We* plateau is best characterized by the model from Equation (1).

Upon impacting a superhydrophobic surface, a droplet undergoes a spreading phase, during which the droplet flattens and spreads over the surface as kinetic energy is converted to surface energy, while partially being lost to viscous dissipation in the bulk. Due to larger impact kinetic energy, spreading is wider and faster at higher *We*, while (if the latter is constant) the influence of droplet diameter is captured by the Reynolds number. Figure 4 shows the dependence of *β*_max_ on *Re*, where one can observe a progressive trend within each *We* level as the droplet diameter is increased (i.e., *Re* is increased). The smaller droplet with higher velocity loses a significantly larger fraction of its initial energy to internal friction compared to the large, low-velocity droplet. Because more energy is dissipated, less kinetic energy remains available to drive the outward expansion of the lamella, resulting in reduced maximum spreading.

The horizontal expansion is not governed by viscous dissipation alone; instead, it is also influenced by gravity as droplet diameter increases (lower *Fr* number). Droplets larger than ~2.7 mm (Bo > 1) result in a gravitationally flattened shape prior to impact. During the spreading phase, gravity provides a more significant supplementary force that actively assists the outward radial expansion of the lamella. This gravitational flattening compounds the effect of reduced viscous dissipation, further increasing *β*_max_, a trend that appears to be even more pronounced at high *We* [17].

If one aims to predict the maximum spreading coefficient, there are several published models available, proposed based on dimensionless scaling or energy conservation. The former correlates the nondimensional numbers (most often *We* and *Re*) that govern droplet rebound with the maximum spreading coefficient by empirically fitted coefficients, whereas the energy conservation method considers that the sum of the droplet kinetic and surface energy before the impact is equal to the surface energy of the droplet at the maximum spread minus the energy losses due to viscous dissipation. Table 1 lists 13 published models from both categories, which were fitted to our data, and the results were evaluated by the coefficient of determination R^2^ and the mean absolute percentage error (MAPE).

Among the empirical models, the best description of data trends was obtained with the model of Jereb et al. [18] (R^2^ = 0.79 and MAPE of 4.7%), while the energy conservation model of Mao et al. [19] (R^2^ = 0.92 and MAPE of 2.7%) was the most accurate overall. When fitting our data to a commonly adopted form of empirical models $a+b\cdot {We}^{c}\cdot {Re}^{d}$, we obtain the following relationship:

(1) $$\beta_{max}=1+0.0314\cdot {We}^{0.515}\cdot {Re}^{0.221},$$

which well describes our dataset (R^2^ = 0.97 and MAPE of 1.4%). The fitted parameters exhibit high statistical significance (*p* < 0.001), with standard errors of 0.0079, 0.027, and 0.043 for the leading coefficient, *We* exponent, and *Re* exponent, respectively. Residual analysis showed that prediction errors are uncorrelated with *Re* and *We* values, meaning that no systematic bias is present in the model.

**Figure 5:**
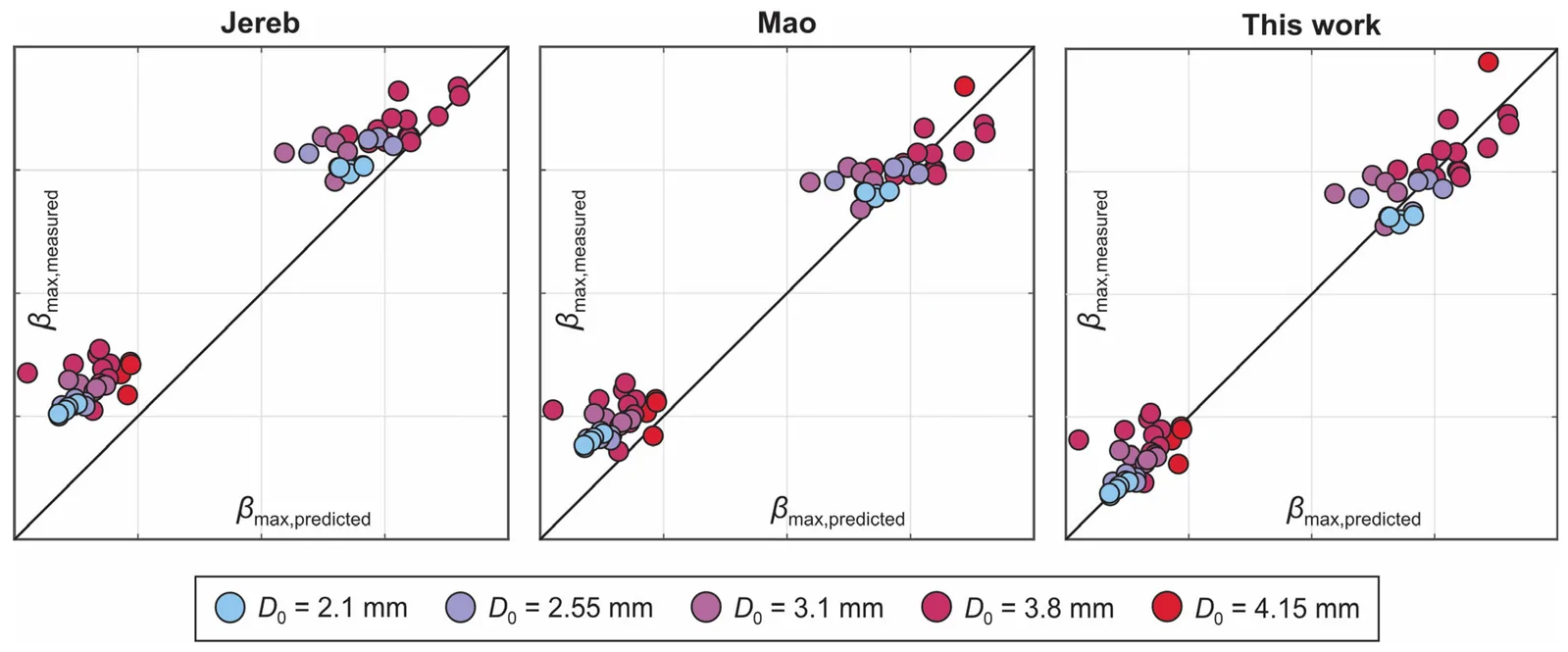
Comparison of the most accurate empirical scaling (Jereb et al.) and energy conservation (Mao et al.) models for predicting the maximum spreading coefficient with the model developed in this work. Both axis ranges in all charts correspond to [1.8 2.6] with a step of 0.2.

Comparing the predictions of the two best performing models with the one of this work (Figure 5) shows that the main improvement appears to be in the prediction of low *We* number data points. There, we assume that our proposed *Re* scaling better captures the events when the liquid properties, namely viscosity, are kept constant (note that both other works also included different liquids). Across all three models, prediction errors appear to be only slightly correlated to droplet diameter, which indicates that the combination of *We* and *Re* is enough to describe the size-related parameters, such as the non-circular shape and the vibrations prior to impact, which are more influential as droplet diameter increases.

**Table 1:** List of models to predict the maximum spreading coefficient and their accuracy.

Empirical scaling models		R^2^	MAPE
Asai et al. [20]	$\beta_{max}=1+0.48\;{We}^{0.5}\times \exp\left[-1.48\;{We}^{0.22}{Re}^{-0.21}\right]\;$	<0	20.1%
Scheller & Bousfield [21]	$\beta_{max}=0.61\;{\left({Re}^{2}\cdot Oh\right)}^{0.166}$	<0	45.0%
Roisman [22]	$\beta_{max}=0.87\;{Re}^{0.2}-0.4\;{Re}^{0.4}/\sqrt{We}$	<0	23.5%
Andrade et al. [23]	$\beta_{max}=1.28+0.071\;{We}^{1/4}{Re}^{1/4}$	<0	20.1%
Jereb et al. [18]	$\beta_{max}=1.1885+0.0397\;{We}^{\pi /6}{Re}^{\pi /18}$	0.79	4.7%
Energy conservation models		R^2^	MAPE
Chandra & Avedisian [24]	$1.5We/Re\beta_{max}^{4}+\left(1-\cos\theta \right)\beta_{max}^{2}-(1/3\;We+4)\approx 0$	<0	29.0%
Pasandideh-Fard et al. [25]	$\beta_{max}=\sqrt{\frac{We+12}{3\;\left(1-\cos\theta \right)+4\;We/\sqrt{Re}}}\;\;$	0.03	11.0%
Mao et al. [19]	$\left[\frac{1}{4}\left(1-\cos\theta \right)+0.2\frac{{We}^{0.83}}{{Re}^{0.33}}\right]\beta_{max}^{3}-\left(\frac{We}{12}+1\right)\beta_{max}+\frac{2}{3}=0$	0.92	2.7%
Ukiwe & Kwok [26]	$\left(We+12\right)\beta_{max}=8+\beta_{max}^{3}\;\left[3\left(1-\cos\theta \right)+4\;We/\sqrt{Re}\right]$	0.64	6.5%
Madejski [27]	$3\frac{\beta_{max}^{2}}{We}+\frac{1}{Re}{\left(\frac{\beta_{max}}{1.2941}\right)}^{5}=1$	<0	57.2%
Yang & Kurabayashi [28]	$\frac{We}{2}=\frac{3}{2}\left[1+\frac{3We}{Re}\left(\beta_{max}^{2}\;ln\beta_{max}-\frac{\beta_{max}^{2}-1}{2}\right)\right]-6$	<0	557.1%
Healy et al. [29]	$\beta_{max}=\beta_{max,KY}{\left(\frac{45}{\theta }\right)}^{0.241}$	<0	381.8%
Aksoy et al. [30]	$3.18\frac{{We}^{0.72}}{{Re}^{0.86}}\beta_{max}^{6.5}+3\left(1-\cos\theta \right)\beta_{max}^{3}-\left(We+12\right)\beta_{max}+8=0$	<0	17.7%

The initial coefficient *a* that equals unity in our model well describes the limiting case of *We* and *Re* numbers equal to 0 (i.e., velocity is 0), where the droplet is motionless on the surface and *D*_max_ equals *D*_0_. In addition, our proposed *We* scaling closely matches that of Jereb et al. [18], even though the latter work included different surfaces, viscosity and a broader parameter span. Since their dataset is publicly available [14], we used Equation (1) to predict all data points, obtaining R^2^ = 0.79 and MAPE 9.1%, which strongly outperformed all of the above models, except the model that was specifically fitted to that dataset. This validation with unseen data that includes completely different surface textures, liquid properties and impact conditions reinforces the generalizability of our model, implying that one can extrapolate it to predict *β*_max_ outside the narrow range where it was developed (note that the unseen dataset features *We* numbers 8–120 and *Re* numbers 9–4400). However, extrapolation beyond the splashing threshold at high *We* remains unverified and should be approached with caution.

### 3.2. Droplet Dynamics During Liquid-Surface Contact

**Figure 6:**
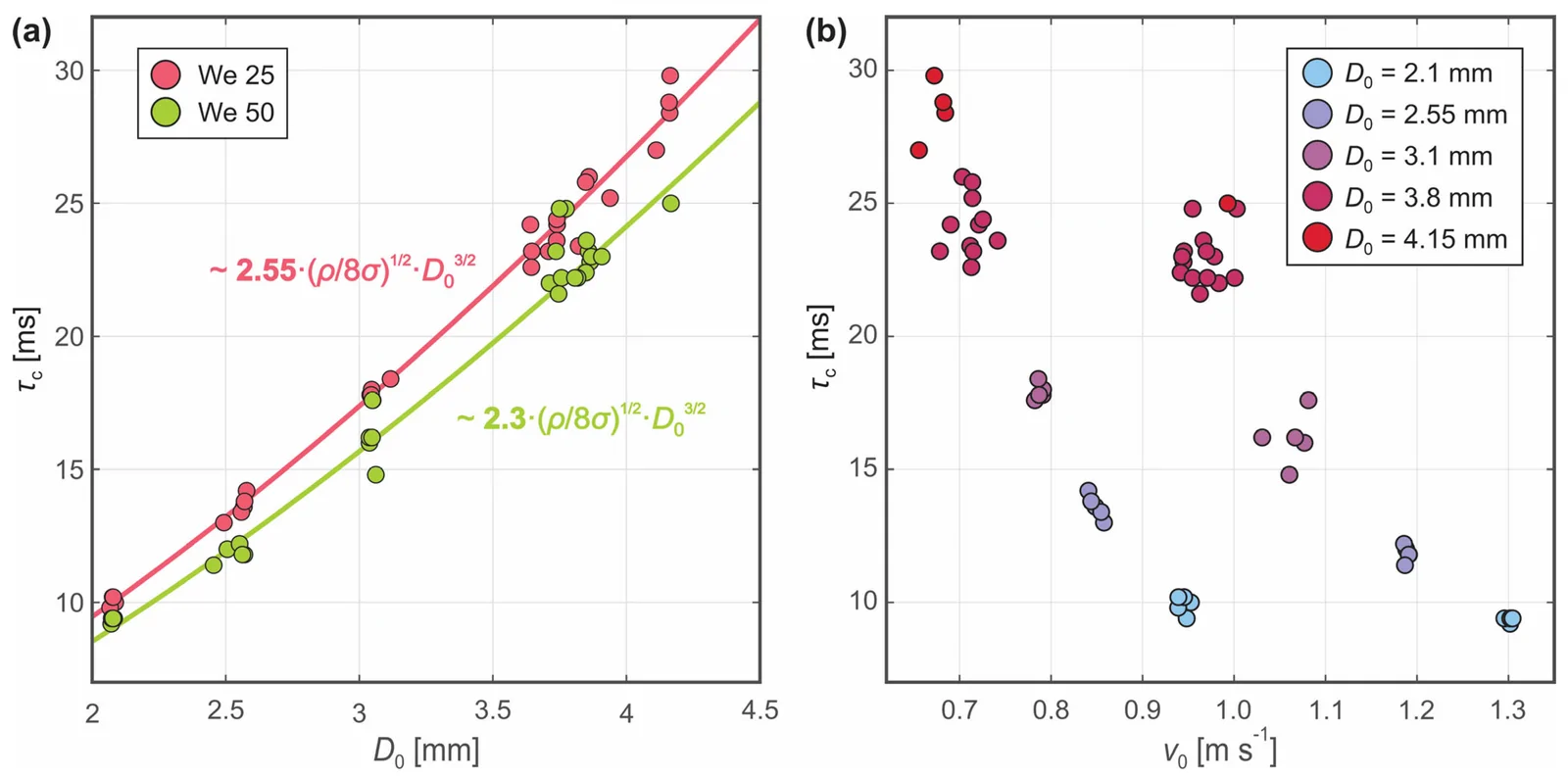
(**a**) Contact time of water droplets of increasing size at two *We* number levels, indicating different inertia-capillary scaling coefficients. (**b**) Contact time of water droplets at different velocities required to achieve *We* of 25 and 50.

Figure 6a shows the expected increase in contact time as the droplet becomes larger, which is clearly visible for both *We* levels. However, we also observe a distinctly different scaling of this progressive trend, with the power law becoming steeper at lower *We*.

When fitting the inertia-capillary scaling ${\left(\rho {R_{0}}^{3}/\sigma \right)}^{1/2}$ over both data groups, we obtain a larger scaling coefficient for the lower *We* number, very close to the value of 2.6 that was originally reported by Richard [6] with *We* values up to 37. Instead, a higher *We* number yields a lower coefficient of 2.3, which resembles the first-order vibration period of a freely oscillating drop, i.e., $\pi /\sqrt{2}$ [31]. This indicates that the contact time of real droplets might not be completely independent of velocity, as shown by Figure 6b. Indeed, the difference between recorded *τ*_c_ of a particular droplet at different velocities appears to increase as the droplet diameter increases. Thus, small droplets with diameters in the order of ~2 mm (which are most common in published literature) exhibit only a minor velocity scaling of contact time, which could easily be overlooked as measurement uncertainty or reproducibility effects. Hence, our data for small 2.1 mm droplets align with the classical velocity-independent baseline, reported by Richard et al. [6]. However, when larger droplet rebounds are considered (at the same *We*), the decreasing trend of contact time becomes clearly visible, with several milliseconds of difference when velocity is increased, in line with recent studies [32,33] which demonstrated that *τ*_c_ exhibits complex deviations under varying impact conditions. Whether this divergence continues over a wider *We* range remains an open question and an option for future work. At significantly higher impact velocities, the onset of splashing will disrupt the uniform retraction phase, whereas at lower velocities (approaching We < 1), the lack of significant spreading would likely mask this observation. A possible explanation for this trend being previously overlooked is the increased scatter of recorded values of contact time of larger droplets due to a larger influence of vibrations and gravitational flattening.

**Figure 7:**
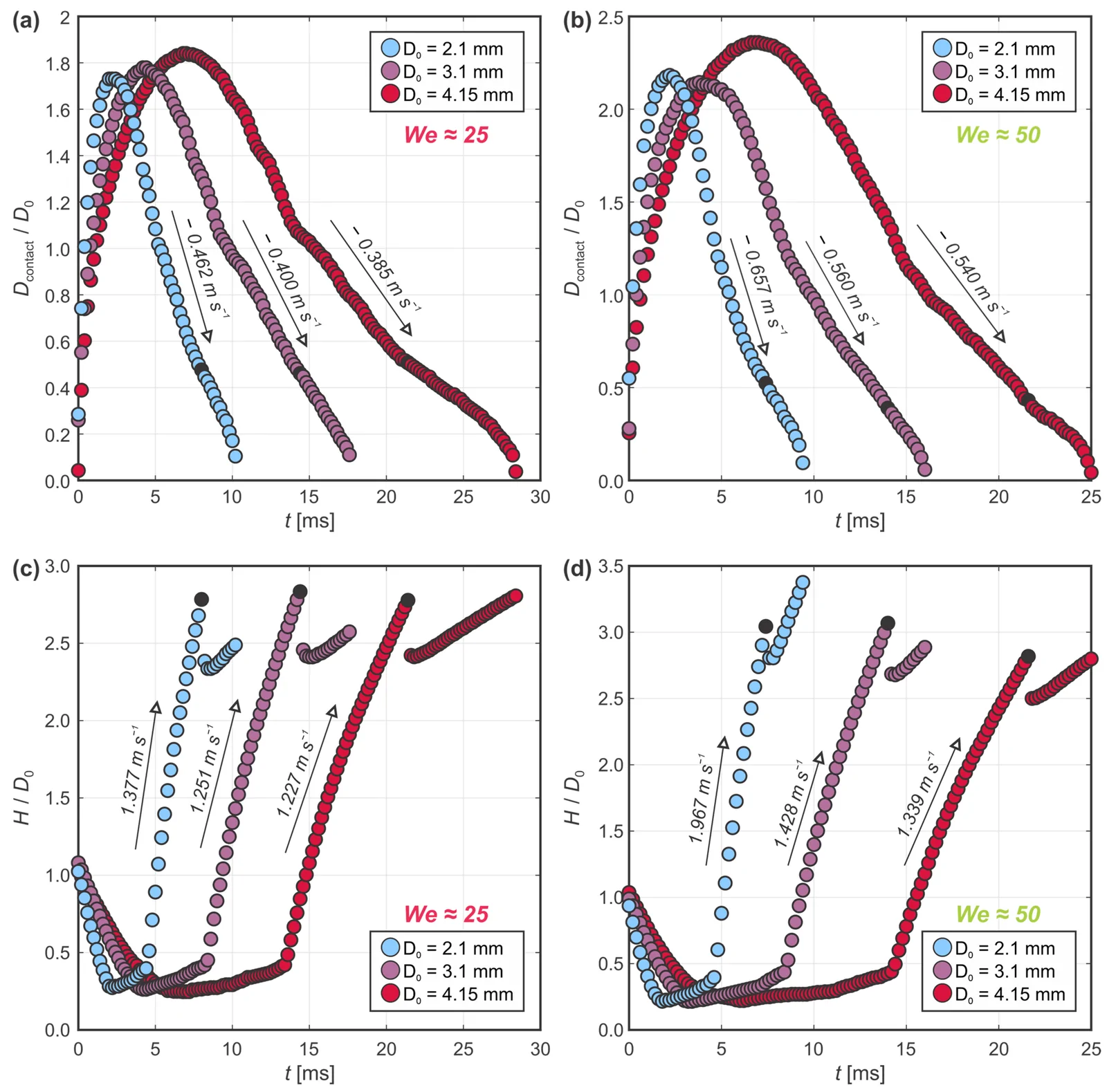
Droplet dynamics during liquid-surface contact. (**a**,**b**) Normalized contact diameter of differently sized water droplets with corresponding average horizontal retraction rate *δD*_droplet_*/δt* at *We* numbers of 25 and 50, respectively. (**c**,**d**) Normalized height of differently sized water droplets with corresponding average vertical propagation rate *δH/δt* at *We* numbers of 25 and 50, respectively. Black scatter points represent the frame of vertical satellite droplet detachment.

To delve deeper into the different values of recorded contact time at a given *We* number level we looked into droplet dynamics of small (2.1 mm), medium (3.1 mm) and large (4.15 mm) drops. Figure 7 shows the normalized contact diameter and droplet height of three experiments per *We*, where the influence of droplet size is clearly visible. At a given *We* number a large drop will experience a larger spread (higher *β*_max_), which will be reached in a significantly longer time, compared to the small droplet. After this timepoint, the rim velocity changes direction, and the droplet retraction begins. With *Oh* values between 0.00177 and 0.00245, the initial stage of retraction is firmly governed by inertia and its horizontal velocity scales as [34]

(2) $$v_{ret,h}\;\sim \;R_{max}\cdot t_{ic}\sqrt{\pi \left(1-cos\theta_{R}\right)},$$

which signals its independence from impact velocity. The latter might be the case for the initial few milliseconds, after which the larger surface energy due to increased flattening at high *We* influences the receding of the droplet, making it quicker compared to the same droplet at lower *We* [15]. We confirm this by observing a steeper falling slope of the normalized contact diameter, which points to a higher average retraction rate (see plot annotations in Figure 7a,b) of a given droplet at a higher *We*. Interestingly, we also observe differences in retraction velocity at constant *We*, implying that droplet diameter plays a role in retraction dynamics. Larger drops retract more slowly, regardless of *We* level, which might be due to more viscous and oscillatory energy losses in a larger liquid bulk. Similar conclusions can be drawn for the vertical velocity (Figure 7c,d), where increasing *We* and *D*_0_ increase the vertical velocity up until the first vertical satellite droplet detaches from the bulk. Faster horizontal movement of the liquid bulk results in a stronger squeeze before the liquid ejects vertically upwards, which yields higher vertical propagation rates of smaller drops with high impact velocity. Once the first vertical satellite drop, comprising liquid with the highest velocity, detaches, the average velocity of the remaining bulk in both directions is reduced. Interestingly, the moment of first satellite ejection occurs around δ*D*_droplet_*/*δ*t* ≈ 0.4–0.5 for all conditions, as depicted by the black circle points in Figure 7a,b. Following this pinch-off, the droplets rebound, with larger drops forming additional secondary droplets while traveling vertically upward. An increased number of satellite drops corresponds to larger droplets and higher *We*—thus, a 4.15 mm droplet at *We* ≈ 50 might form up to 6 satellites. The time between consecutive pinch-offs appears to reduce with increased liquid mass velocity. Thus, a smaller and faster drop might eject fewer satellites, but do so in quick succession.

**Figure 8:**
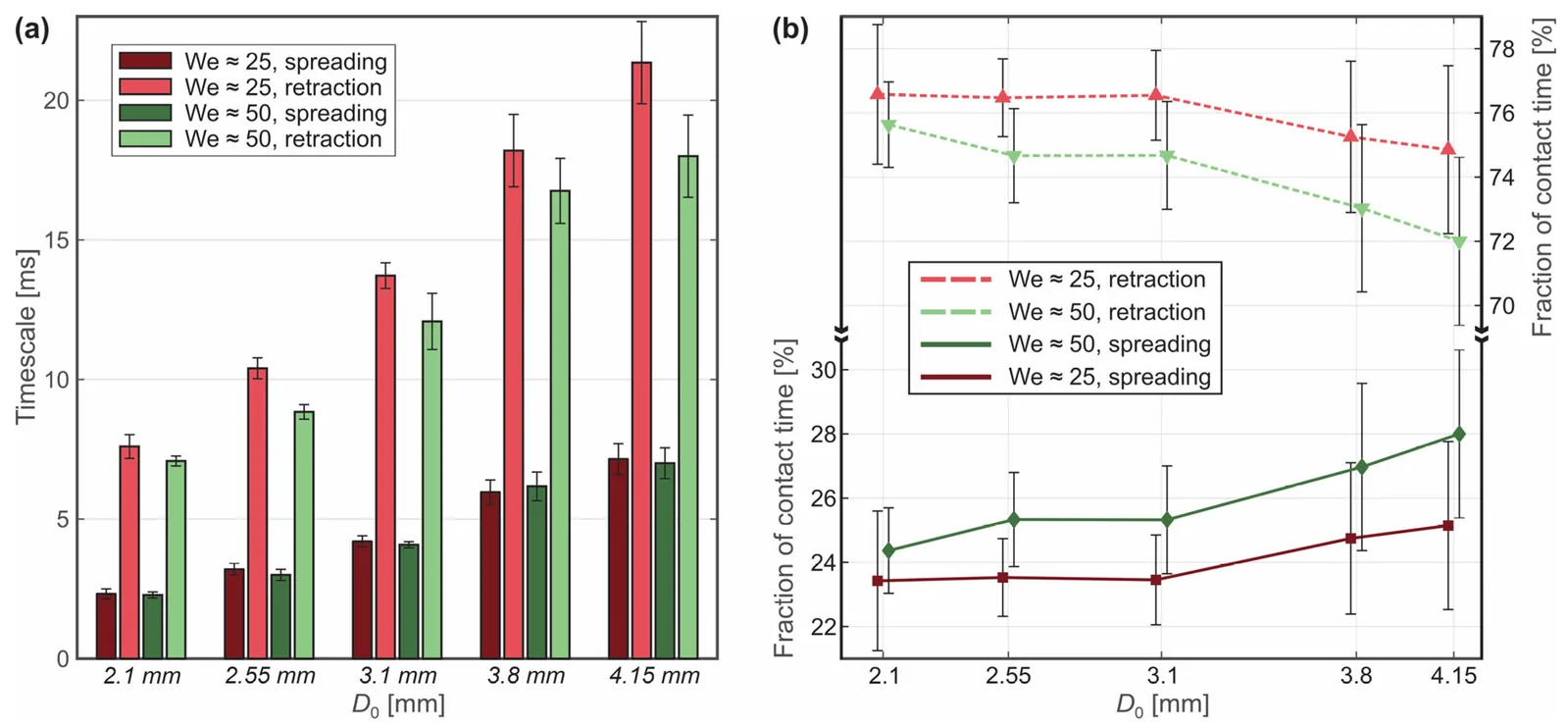
(**a**) Average droplet spreading and retraction timescales. Error bars denote the standard deviation of measurements. (**b**) Average fraction of contact time needed for droplet spreading and retraction. Error bars denote the standard deviation of measurements.

To further distinguish rebound dynamics of different drops at the same Weber number, attempting to explain the observed difference in inertia-capillary scaling (Figure 6a), we compared the retraction and spreading timescales of all experiments. Figure 8a shows that an increase in droplet diameter at fixed *We* induces a longer retraction and spreading timescale, resulting in a larger contact time. Interestingly, the rate of this change is not the same when comparing *We* levels, indicating that the relative importance of the two rebound phases might be changing. Thus, we compare the fraction that spreading and retraction occupy in the whole contact time, plotted for both *We* groups per droplet diameter (Figure 8b). Results demonstrate that for lower *We*, the influence of droplet diameter on rebound dynamics is much less pronounced, with smaller droplets exhibiting the same ratio of spreading and retraction. Once the droplet diameter is further increased (3.8 mm and 4.15 mm), spreading takes a slightly larger portion of time. This phenomenon is even more pronounced at higher *We*, where the time fraction of spreading immediately begins to increase with the increase in droplet diameter. Thus, when the droplet diameter increases by a factor of ~2, spreading contributes 4% more to contact time, while the contribution of retraction is appropriately reduced.

We can rationalize this by considering the *Re*, which scales proportionally to the square root of droplet diameter when *We* is fixed. Thus, larger droplets (with lower velocity) experience reduced relative viscous dissipation within the bulk liquid, meaning that they continue to expand laterally for a longer duration and ultimately reach a higher *β*_max_. The absolute duration of the spreading phase can be loosely approximated as the ratio of the maximum spread radius to the impact velocity. Substituting $v_{0}=\sqrt{We\cdot \sigma /2\rho R_{0}}$ for a fixed Weber number yields:

(3) $$t_{s}\approx \frac{\beta_{\max}R_{0}}{v_{0}}=\beta_{\max}\sqrt{\frac{2}{We}}\sqrt{\frac{\rho R_{0}^{3}}{\sigma }}=\beta_{\max}\cdot \sqrt{\frac{2}{We}}\cdot t_{ic},$$

Unlike spreading, retraction is driven primarily by capillary forces attempting to minimize the surface area and scales closely with the inertia-capillary timescale. When a larger droplet spreads to a higher *β*_max_, its liquid volume is distributed over a wider area, which creates a thin liquid lamella. The retraction of this liquid film is governed by the Taylor–Culick edge velocity, which dictates that thinner lamellas retract faster. Thus, retraction velocity is driven by capillary pull and scales inversely with lamella thickness *h* [35], in accordance with $v_{r}=\sqrt{2\sigma /\rho h}$. Figure 7c,d clearly shows that smaller droplets at fixed *We* produce a thinner lamella (lower *H*, when multiplied by *D*_0_), which explains the recorded higher retraction rates. Additionally, the rim of a smaller drop needs to travel less since *D*_max_ is smaller, meaning that in absolute units the retraction occurs much faster (Figure 8a). However, dimensional analysis shows that, contrary to spreading velocity, retraction speed does not depend on *β*_max_. Assuming that a maximally spread droplet approximates a flat cylinder, volume conservation dictates that *h* is geometrically linked to the initial droplet radius and the maximum spreading radius as $h\approx 4R_{0}/3\beta_{\max}^{2}$. Substituting this absolute lamella thickness into the expression for the Taylor-Culick edge velocity yields a retraction velocity that is directly proportional to the spreading coefficient $v_{r}\approx \beta_{\max}\sqrt{3\sigma /2\rho R_{0}}$. The characteristic retraction time $t_{r}$ can be approximated by the ratio of retraction distance and velocity, revealing its independence from the maximum spreading coefficient:

(4) $$t_{r}\approx \frac{0.5\beta_{\max}D_{0}}{\beta_{\max}\sqrt{3\sigma /\rho D_{0}}}=\frac{1}{2\sqrt{3}}\sqrt{\frac{\rho D_{0}^{3}}{\sigma }}=\sqrt{\frac{2}{3}}t_{ic},$$

This demonstrates that the increased radial distance that a widely spread droplet must travel is compensated by the accelerated capillary retraction of its proportionally thinner lamella.

As shown in Figure 4, increasing the droplet diameter at a fixed *We* yields a higher *Re*, which reduces relative viscous dissipation and allows the droplet to achieve a higher *β*_max_. Because spreading time scales with *β*_max_ while retraction time remains strictly bound to the inertia-capillary timescale, the relative duration of the spreading phase inherently lengthens for larger droplets. Consequently, this extension of the spreading phase forces the relative fraction of time spent retracting to decrease, explaining the divergence of trends observed in Figure 8b. We can show that this size-induced difference in the relative importance of spreading and retraction is the reason behind the different scaling of contact time shown in Figure 6. With $\tau_{c}=t_{s}+t_{r}$, one can write the inertia-capillary scaling coefficient to be approximately proportional to $\beta_{\max}\cdot \sqrt{2/We}+\sqrt{2/3}$, which explains the changed scaling when *We* number increases. While this theoretical derivation captures the shifting temporal proportions of spreading and retraction, comparing the theoretical values to experimental data reveals a discrepancy. The experimental spreading timescale aligns reasonably well with the derived inertial prediction; however, the theoretical retraction time ($t_{r}\approx 0.82t_{ic}$) severely underestimates the experimentally observed values, which scale closer to $1.82t_{ic}$. This is attributed to the fact that Equation (4) derivation relies on one-dimensional kinematics and constant velocities, ignoring three critical energy sinks that dictate droplet retraction: (i) rim mass accumulation, (ii) momentum redirection in the vertical direction, and (iii) contact line friction driven by surface non-uniformity and contact angle hysteresis. While the measured hysteresis of 6.1° is relatively low, it still induces an impeding force that scales with the contact line perimeter. Consequently, droplets achieving a larger *β*_max_ experience greater pinning forces. This dictates that the actual retraction velocity is hindered more than the ideal case predicts and that retraction time retains a weak dependence on the spreading extent, which contributes to the substantial gap between theoretical and experimental timescales. Nonetheless, we can infer that the redistribution of relative timescales would be even more pronounced at higher *We* or larger droplets than the ones in this study, which warrants further investigation in the future.

## 4. Conclusions

This study systematically decouples the effects of water droplet diameter and impact velocity at fixed Weber numbers (25 and 50) to evaluate the hydrodynamic similarity during droplet rebound on superhydrophobic aluminum surfaces.

At a fixed *We*, the maximum spreading coefficient *β*_max_ increases progressively with *Re*. Smaller droplets with higher impact velocities experience greater dynamic pressures and higher relative viscous dissipation, which restricts their lateral expansion. A modified empirical scaling model was proposed that achieves excellent agreement with our experimental data (R^2^ = 0.97, MAPE = 1.4%) and good initial generalizability when tested against an independent literature dataset featuring different fluid properties, surface characteristics, and Weber numbers. To improve the suggested correlation, further testing is required across a wider range of *We* and *Re* numbers.

Furthermore, while classical models assume that contact time is independent of impact velocity in the inertia-dominated regime, we demonstrate the opposite, with inertia-capillary scaling reducing with Weber number. This difference is especially pronounced for larger droplets (e.g., *D*_0_ > 3 mm), where contact time decreases by several milliseconds as impact velocity is increased. We explain this phenomenon with a redistribution of rebound phases—as droplet size increases, spreading occupies a larger fraction of the total contact time, whereas the relative duration of the retraction decreases. We rationalize this shift through a dependency of spreading timescale on the maximum spreading diameter, while the retraction duration remains strictly bound to the inertia-capillary time. Extending this analysis to a wider spectrum of Weber numbers, particularly approaching the splashing threshold, will be considered in our future work, together with the interplay of contact angle hysteresis and droplet size.

This work provides insights into engineering robust biomimetic technologies, such as anti-icing or self-cleaning surfaces, that optimize droplet-surface interactions under diverse real-world conditions. For developing surfaces that rely on mimicking the efficient water-shedding properties of a biological surface, we demonstrate that accurately replicating this natural adaptability across different scales requires accounting for size-dependent deviations in droplet spreading and retraction timescales.

## Data Availability

The authors confirm that the data supporting the findings of this study are available either within the article or from the corresponding author upon reasonable request.

## Abbreviations

The following abbreviations are used in this manuscript:

EDS	Energy-dispersive X-ray spectroscopy
HDPA	Hexadecyl phosphonic acid
IPA	Isopropyl alcohol
LED	Light-emitting diode
MAPE	Mean absolute percentage error
SEM	Scanning electron microscopy
UV	Ultraviolet
